# Microbial Composition Dynamics in Peloids Used for Spa Procedures in Lithuania: Pilot Study

**DOI:** 10.3390/ijerph21030335

**Published:** 2024-03-12

**Authors:** Marija Kataržytė, Lolita Rapolienė, Greta Kalvaitienė, Rafael Picazo-Espinosa

**Affiliations:** 1 Marine Research Institute, Klaipėda University, University Avenue 17, 92295 Klaipėda, Lithuania; greta.kalvaitiene@ku.lt (G.K.); rafael.picazo.espinosa@ku.lt (R.P.-E.); 2 Faculty of Health Sciences, Klaipeda University, H. Manto Str. 84, 92294 Klaipėda, Lithuania; lolita.rapoliene@ku.lt

**Keywords:** sapropel, long-read sequencing, *Escherichia coli*, clostridia

## Abstract

Despite peloids’ acknowledged therapeutic and cosmetic potential, there remains a limited understanding of their microbial diversity and dynamics, especially concerning beneficial and non-beneficial microorganisms under different heating conditions. Our study employs both cultivation and metagenomic methods to assess the microbiota of peloids, focusing on lake sapropel and peat under heating conditions recommended for external application and safety assurance. By applying microbial indicators specified in national regulatory documents, we found that all peloids reached thresholds for sulphite-reducing clostridia and colony-forming units. Each peloid exhibited a distinctive bacterial composition based on metagenomic analysis, and temperature-induced changes were observed in microbial diversity. We identified beneficial bacteria potentially contributing to the therapeutic properties of peloids. However, the same peloids indicated the presence of bacteria of human faecal origin, with a notably higher abundance of *Escherichia coli*, pointing to a potential source of contamination. Unfortunately, it remains unclear at which stage this contamination entered the peloids. The findings underscore the importance of monitoring and controlling microbial aspects in peloid applications, emphasising the need for measures to prevent and address contamination during their preparation and application processes.

## 1. Introduction

Healing mud, also known as therapeutic mud or peloid, is a natural substance composed of minerals, organic matter, and water. The International Society of Medical Hydrology defines peloids as “substancSes formed under the geological influence of natural conditions that are mixed with water and used in medical practice in the form of baths or local procedures” [1]. The recent definition proposed by [2] defines peloid as “a maturated mud or muddy dispersion with healing and/or cosmetic properties, composed of a complex mixture of fine-grained natural materials of geologic and/or biologic origins, mineral water or seawater, and commonly organic compounds from biological metabolic activity”. Peloids are the subject of numerous studies in various fields, such as geology, cosmetology, materials science, pharmacy and medicine [3,4,5]. They possess multiple therapeutic properties and have been used in traditional medicine and spa treatments for centuries. Their use by humans for medicinal and wellness purposes is probably as old as mankind. The first references to the medical use of peloids were recognised in Ancient Egypt in the 7th century BC [6,7].

While peloids’ composition and properties may vary depending on their source, several microbiological properties are commonly associated with their healing effects, such as antimicrobial activity, anti-inflammatory effects, probiotic properties, detoxifying, moisturising and nourishing effects and wound healing properties. They might contain natural antimicrobial compounds, such as humic and fulvic acids, that can inhibit the growth of certain microorganisms. These antimicrobial properties help prevent or reduce the risk of infections when the mud is applied to the skin. Some peloids may contain beneficial bacteria or probiotics that can positively influence the skin microbiota [8,9]. These probiotic bacteria can help restore the natural balance of microorganisms on the skin’s surface, promoting skin health. Based on previous research, it was identified that *Actinobacteria* or *Cyanobacteria* present in mud could produce substances with antimicrobial activity, and *Aquaphilus* and *Pelobacter* have been identified for their anti-inflammatory properties [8,9]. Some microorganisms present in peloids can break down minerals, making them more accessible to the skin. Metabolic activities of sulphur- and iron-reducing bacteria like *Deferribacterales* could generate sulphur compounds, which may play a role in skin disease management [10]. Certain microbes may assist in detoxifying the skin by breaking down harmful substances or removing toxins [11] from peloids.

The efficacy and specific properties of peloid can vary depending on its source, preparation and application methods and individual characteristics, such as the extent of body coverage or the body coverage area, individual skin characteristics, the duration of the treatment course, and the number of procedures. Some microbiological issues, such as pathogenic bacteria contamination and fungal or parasitic infections, can be associated with using peloids [6]. Natural peloids sourced from peatlands or lake sediments, in addition to autochthonous microorganisms, may harbour an external microbiota, which could potentially contain undesirable pathogenic organisms, many of which originate from human activities. If the peloids are not properly sourced, processed, or stored, they can contain pathogenic bacteria such as *Escherichia coli*, *Staphylococcus aureus*, or *Pseudomonas aeruginosa*, which can cause infections if they come into contact with open wounds or broken skin [12]. Fungi, including various species of moulds and yeasts, can also be present in peloids. These organisms can cause skin infections, such as dermatitis or fungal nail infections (onychomycosis). 

Lithuania is known for health and medical spa treatment (legally recognised health establishment under medical supervision with natural healing waters and other natural resources). In Lithuania, the healing properties of Druskininkai, Birštonas mineral water, and peloids have been known since the 13th–16th centuries [13]. Druskininkai and Birštonas are located in eastern Lithuania and have a resort status due to their wellness spa services. The mineral waters used in spa procedures are derived from Triassic and Cretaceous deposits. The mineralisation of these waters is usually low to medium (2–35 g/L NaCl), with a neutral pH (7–7.5) [14]. Lithuanian spa facilities use internal natural resources of sapropel from the lakes, as well as clay or peat from peat quarries mixed with mineral water for the treatment of connective tissue, muscles and skeleton, nervous, digestive, urogenital, respiratory, endocrine, ear, nose and throat, and skin conditions. Most spa facilities employ peloids occurring in situ, either in their extracted form for procedures or mixed with mineral water. Using those peloids decreases pain and muscle tone, increases the amplitude of joint movements, suppresses inflammation, improves psycho-emotional condition [15], and changes physiological processes [16].

There is no uniform standard to regulate the inspection of peloids used for external applications, such as thresholds of heavy metals and harmful microorganisms. Few countries, such as Germany, France, or Cuba, follow national microbiological regulations [6]. In Lithuania, specific threshold values for microorganisms such as *Escherichia coli*, *Staphylococcus aureus*, *Pseudomonas aeruginosa*, *Salmonella*, sulphite-reducing clostridia, colony-forming units of bacteria, and eggs and larvae of pathogenic worms and protozoa are used to ensure the safe use for the spa activities [17]. Although specific microbiological criteria for therapeutic peloids exist as defined by ISO 21426:2018 [18], this material regarding beneficial and non-beneficial microorganisms is under-reported [19,20], mainly due to the limitations of traditional cultivation methods. Next-generation sequencing techniques might provide a more comprehensive view of microbial diversity because they can detect various microorganisms (bacteria, archaea, fungi, and viruses), including those that are non-cultivable or in low abundance [21].

Peloids are supposed to be sourced from clean and uncontaminated environments to minimise the risk of microbiological contaminants associated with peloids. It should also undergo appropriate processing, such as sterilisation or heat treatment, to eliminate or reduce the presence of harmful microorganisms. Additionally, spas or facilities offering peloid treatments should maintain strict hygiene practices and regularly monitor the quality and safety of it. Each spa has its peloid storage facilities or receives it from a supplier. Peloids can be prepared and stored in containers, kept dry, and mixed with mineral water before the procedure or matured in containers. There are no requirements for peloid collection or storage conditions; therefore, how the microbiological composition of peloids changes through those stages has yet to be discovered. Before application, it should be heated to between 50 °C and 55 °C to ensure safe use for external applications [10]. This temperature might affect the microbiological composition of peloids, affecting both the potential pathogens, if present, and the beneficial community.

The aim of the study was to comprehensively investigate the microbial properties of peloids, specifically lake sapropel and peat, utilised in spa treatments in Lithuania. The focus was on understanding the microbial diversity and dynamics, distinguishing between beneficial and non-beneficial microorganisms, including under various heating conditions recommended for safe external application.

## 2. Materials and Methods

**Figure 1:**
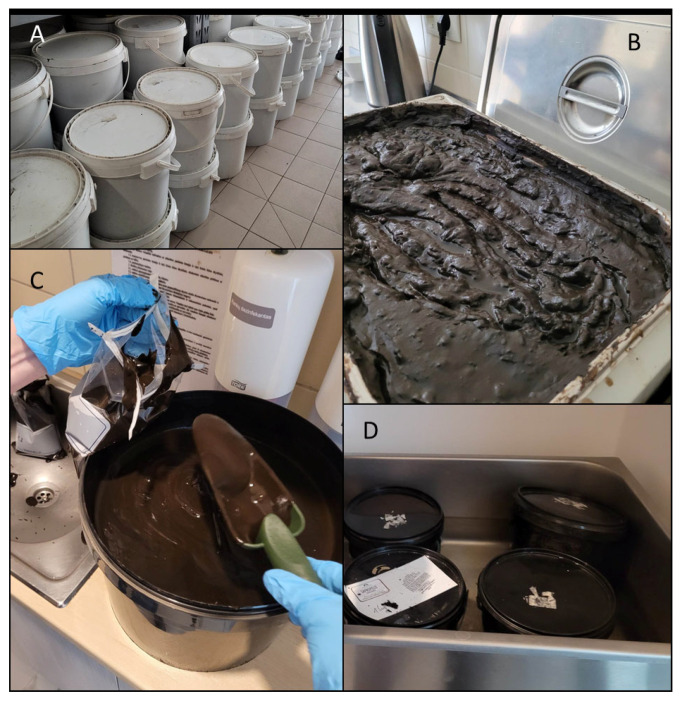
The storing of peloid in the spa facility (**A**), different approaches of peloid heating between 50 and 55 °C before the application in spa facilities (**B**,**D**), and collection of peloid samples for the analyses (**C**).

Peloid samples were taken from four different spa facilities. The supplied peloid sources were sapropel from three different lakes and one peat reservoir, allocated at various places in Lithuania. Ready-to-use peloid samples (P1–4) were collected aseptically at each facility in July–August 2023 in a sterile plastic bag (up to 1 L), labelled, and transported in a cooler to the lab (Figure 1). 

In the lab, samples from each place were divided into three parts in triplicates to imitate the preheating procedures used in the spa before the peloid application. One set of samples was analysed without applying preheating (initial sample). One set of samples was placed in the autoclavable, sterile bags (up to 100 g) and kept in the water bath at 40 °C for half an hour (following the temperature range from 40 to 42 °C, suggested as optional for application on the skin by HN 126:2010), and one set of samples was kept in the water bath at 50 °C for temperature (according to temperature range from 50 to 55 °C suggested to ensure microbiological safety by HN 126:2010). From each sample set, the following analyses were performed: (a) total coliforms and *E*. *coli* amount; (b) presence of *Candida albicans* and *Aspergillus niger*; (c) metagenomic for bacteria diversity and relative abundance.

Ten grams of peloids (dry wet weight) were extracted with 10 mL of sterile distilled water using orbital shaking for 30 min at 100 rpm. The extract was then plated (0.2 mL) in triplicates per media (Sabouraud’s Dextrose agar (SDA) and Mycosel agar) [22] for fungi cultivation. Plates were incubated for five days in SDA and 21 days in Mycosel agar at 27.5 ± 0.5 °C. The *Aspergillus* and *Candida*-alike colonies were examined for identification at the species level. However, no cultured colony showed the *Aspergillus* or *Candida*-alike colonies or morphological structures.

The total coliforms (TC) and *E. coli* quantities were determined using the Colilert-18 (Idexx Laboratories, Westbrook, ME, USA) method. Next, 10 g of peloids was taken, mixed with 100 mL of PBS, shaken for 2 min at 100 rpm, and then allowed to stand. The supernatant was extracted and analysed according to the manufacturer’s instructions. The trays were incubated at 37 °C for 18–24 h. The obtained quantities were later recalculated to express them as MPN/g.

The DNeasy PowerSoil Kit (Qiagen, Singapore) was used to extract DNA from the peloids (0.25 g). Extraction of DNA was performed in triplicate from one sample. Extracted DNA was kept at −20 °C before being used to study bacterial or fungal communities.

Primers of 16S rRNA gene V1-V9 (27F-AGAGTTTGATCCTGGCTCAG; 1492R-GNTACCTTGTTACGACTT) were used to amplify the gene fragments for each peloid sample for bacterial community analysis. Libraries were generated from purified PCR products using the SMRTbellTM Template Pre Kit (PacBio). High-throughput libraries were sequenced on the PacBio Sequel platform. PCR amplification, library preparation and sequencing were performed by Novogene (Cambridge, UK) providing clean sequences.

The obtained demultiplexed PacBio fastq sequence files were imported into qiime2 (2023.7 version) as single-end sequences with quality, and FastQC and the qiime visualisation tool were used to inspect their quality (https://www.bioinformatics.babraham.ac.uk/projects/fastqc/, https://view.qiime2.org/, accessed on 31 January 2024) [23,24]. To remove PCR primers and possible remaining sequencing adapters, the *Cutadapt* plugin of qiime2 was used [25]. Based on the trimmed sequence lengths, denoising with the *dada2* plugin was performed with a preserved length of 1404 nucleotides [26]. The obtained representative sequences were used as input in the taxonomic classification step. The Amplicon sequencing variants (ASVs) were compared with the Silva 138 database [27] at a 99% confidence level using a Naive Bayes pre-trained classifier and the *sklearn* machine learning algorithm [28,29]. The classification results were used to generate taxa bar plots. To ease the analyses of alpha and beta diversity, the *align-to-tree-mafft fasttree* pipeline from the q2-phylogeny plugin was used to build a rooted phylogenetic tree (by fast Fourier transform) [30].

The obtained phylogenetic tree, merged with metadata containing the sample names, was used as input for the alpha and beta diversities study. Alpha rarefaction curves were obtained with the qiime *diversity alpha-rarefaction* plugin. The maximal rarefaction depth was 727, and the frequency number of the sample with the highest sequence count was P3.I. The alpha rarefaction curves of the majority of samples approached or reached the plateau even at lower sequencing depths, indicating that the sequencing effort allowed to account for the great majority of the (genetic) (bio-) diversity of the samples. The same tree was used as input for the beta diversity study using the qiime *diversity beta-rarefaction* plugin, with clustering method upgma (distances matrix based on weighted UniFrac, 250 maximal sampling depth with 10 iterations and Spearman correlation method) [31]. The beta rarefaction results were visualised using the qiime2 visualisation tool (https://view.qiime2.org//, accessed on 31 January 2024) and Emperor PcoA viewer (https://biocore.github.io/emperor//, accessed on 31 January 2024). The clustering tree of the samples was visualised using Mega 11 [32]/figtree [33]/ETE3 etetoolkit tree viewer [34]/iTOL [35]. To have a more precise and robust view of the abundance of the assigned taxa, the ASVs representative sequences table obtained at the *dada2* denoising step was conditionally filtered to retain the ASVs with a relative abundance of ≥1% in at least one of the samples. The filtered table was collapsed to the genus level (Table S1) and used as an input to build a heatmap with the *feature-table heatmap* plugin. The obtained heatmap was based on normalised relative abundances of the different genera (Braycurtis distances matrix, weighted method). To focus on the most abundant taxa across all the samples, more restrictive filtering was applied to the ASVs table, retaining only those sequences appearing ≥ 8 times in at least one sample; therefore, in this additional heatmap, only the most abundant 26 genera were analysed. Still, all the samples were included [36].

## 3. Results

### 3.1. Microbiological Properties of Peloids and Their Dynamic during Preheating

The peloids used in this study (Table 1) were assessed for microbiological parameters according to LT Hygiene norm 126:2010 (Table 2). All parameters except sulphite-reducing clostridia for all samples and colony-forming units for samples P3 and P4 did not reach the thresholds. The highest numbers of sulphite-reducing clostridia were found in samples P1 and P2. 

In experimental analysis with preheating of samples (40 °C and 50 °C), the following microbiological parameters were assessed: (a) total coliforms and *E*. *coli* amount and (b) presence of *Candida albicans* and *Aspergillus niger*. Targeted fungi species were found in none of the analysed samples. Total coliforms and *E. coli* were present in samples P2 and P4; however, they did not reach the thresholds (Table 3).

**Figure 2:**
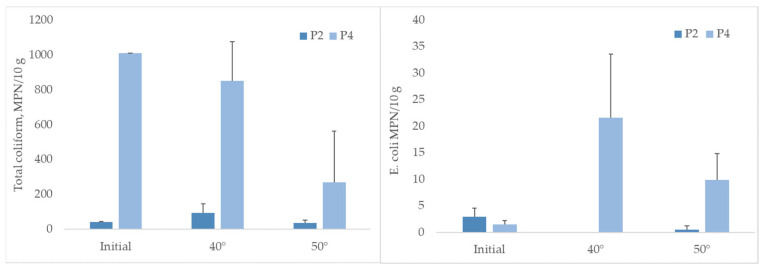
The total coliform and *E. coli* abundances (average ± STDEV) at different temperature conditions in samples P2 and P4.

Analysing the total coliform and *E. coli* numbers in 10 g of peloids, we found some changes under different temperature conditions (Figure 2). In sample P4, when there were initially higher amounts of total coliform, the abundance declined by increasing the temperature. *E. coli* abundances increased 14 times at 40 °C and six times at 50 °C compared to abundances at the initial conditions.

### 3.2. Bacterial Diversity and Dynamics in Peloid Samples

**Figure 3:**
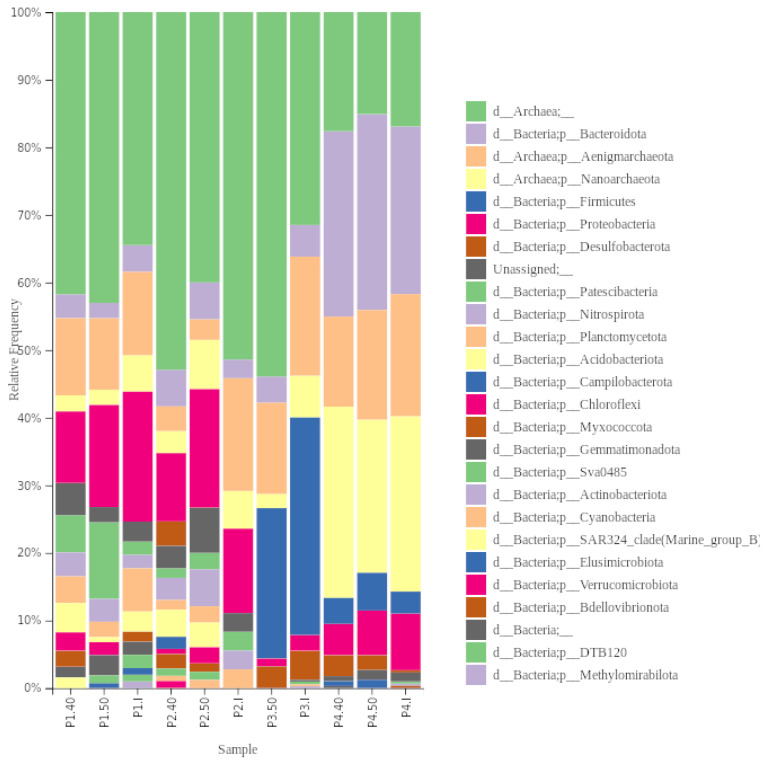
Taxonomy barplot of the samples, based on the relative abundances of the different taxa assigned by comparison of the denoised sequences with the Silva database (version 138).

In our studied samples, we identified both archaea and bacteria. Archea constituted a significant part of samples P1, P2 and P3, but it will not be analysed further (Figure 3). In total, 20 different phyla of bacteria were identified in our samples. 

In the 11 samples analysed, 60,884 reads and 4577 ASV were obtained. The number of reads ranged from 4486 to 6071 with an average read length of 1456 bp, while the number of features in each sample ranged from 9 to 727 (Figure S1).

Regarding bacteria, Proteobacteria was the dominant phylum in the initial samples P1 and P2, constituting 42.8% and 53% relative abundance of total bacteria abundance, respectively. In sample P3, Firmicutes (73%) dominated, while Bacteroidota was dominant in sample P4 (65.7%). Planctomycetota were the second most dominant (14.3%) phylum in sample P1, while in sample P2, Bacteroidota, Nitrospirota, Paetiscibacteria, and Planctomycetota had the same proportion (12.3%), and in P3—Bacteroidota (10.5%), in P4—Proteobacteria (22.2%).

The composition at the phyla level shifted after the preheating of samples. The relative abundance of Bacteroidota and Proteobacteria, identified in all samples, changed differently. The relative abundance of Bacteroidota stayed similar or increased at 40 °C and remained similar at 50 °C compared to the initial relative abundance. The relative abundance of Proteobacteria decreased after preheating but was higher at 40 °C than at 50 °C. Desulfobacteriota was not found in sample P2 but was present at 40 °C (9.8%), and increased in sample P4 (I—<1%, 40 °C—7.8%, 50 °C—5%).

**Figure 4:**
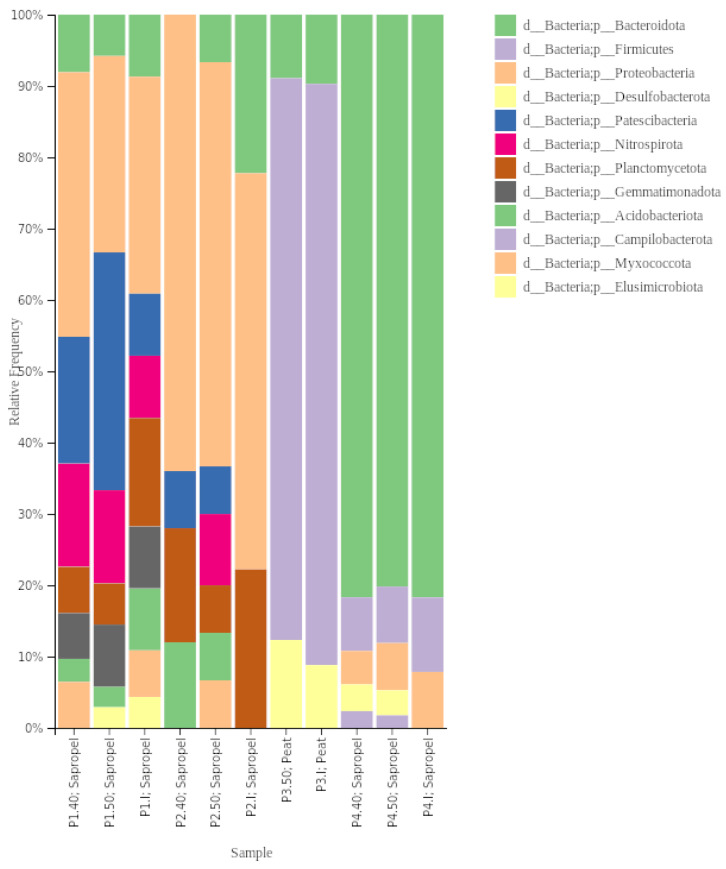
Taxonomy barplot of the different bacterial taxa with ≥ 1% relative abundance in at least one of the samples at the phyla level.

Chloroflexi was not found in any initial samples, albeit it was present in samples P1 and P2 after preheating at both temperature regimes. The most significant shift in composition was observed in sample P2, where, after preheating at 40 °C and 50 °C, eight phyla and five phyla that were not identified in the initial sample were found, respectively (Figure 4). Acidobacteriota was the most abundant phylum in both temperature regimes from the phyla not found in the initial sample, consisting of 10% and 8.5%, respectively. No shift in phyla level due to temperature was observed in the sample P3.

**Figure 5:**
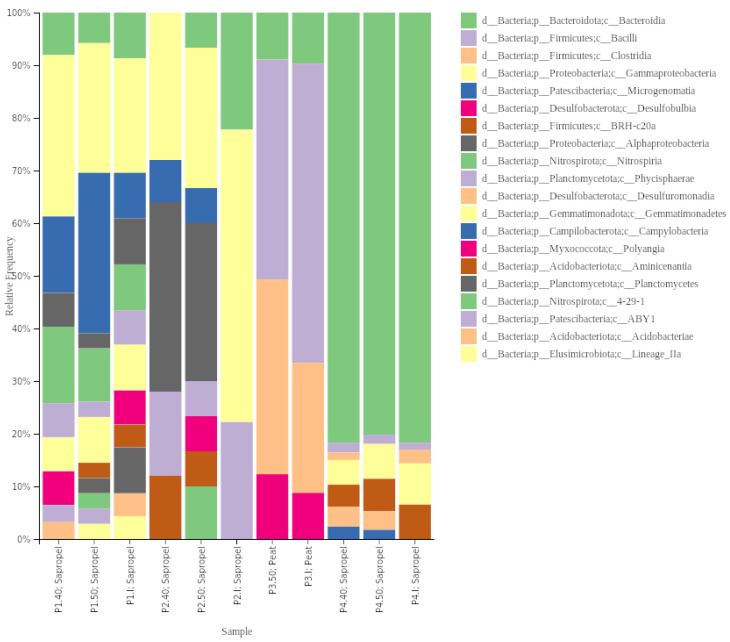
Taxonomy barplot of the different bacterial taxa with ≥1% relative abundance in at least one of the samples at the class level.

In the initial sample of P1 and P2 on the class level, *Gammaproteobacteria* dominated 21.7% and 55.6% relative abundance, respectively (including an order for the *Burkholderiales*, formerly known as *Betaproteobacteria* and now within the *Gammaproteobacteria*). In sample P3—*Bacilli* (56.8%) and sample P4 *Bacteroidia* (81.7%) were the dominant classes (Figure 5). In sample P1, at 40 °C, the relative abundance of *Gammaproteobacteria* increased to 30.7% and, at 50 °C, the dominant class was *Microgenomatia* (30.4%). In sample P3 at 50 °C, the relative abundance of *Clostridia* (37%) and *Desullfobulbia* (12.3%) increased, while in sample P4—at both temperature regimes—the dominant class was *Bacteroidia* (80.2–81.7%), and representatives of *Desulfurumonadia* (3.5–3.8%) and *Campylobacteria* (1.8–2.4%) appeared.

When analysing sequencing data of the 26 most abundant genera, different bacterial compositions were revealed in samples taken from various places. In the sample P1, nine genera were identified as abundant. Higher relative abundance was observed of *Candidatus Woykebacteria* and the genera of the *Methylophilaceae* family at 50 °C and of *Nitrospira* (represented by *Nitrospira lenta*) at 40 °C. 

**Figure 6:**
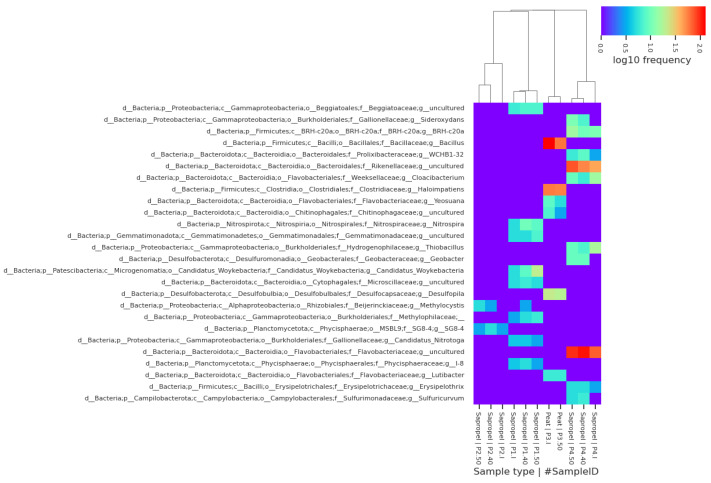
Heatmap of the 26 most abundant genera present in at least one of the samples.

In the sample P2, the genus of *Planctomycetota* was identified in all treatment conditions (Figure 6). *Methylocytis* (represented by *M. parvus*, *M. echinoides*, *M. bryophila*, *M. silviterrae*) was found in all conditions of P2 and in sample P1 at 40 °C.

In sample P3, as the most abundant *Bacillus* (representative *Mesobacillus thioparans* and *M. subterraneus*), *Haloimpatiens* genera and *Desulfopila interna* were identified. In sample P4, the highest relative abundance was observed for uncultured *Rickenellacea* and *Flavobacteriaceae*, whose relative abundance increased in preheated samples.

In the analysed samples, we identified several species that could be of concern due to the environment where they are usually found or their potential pathogenicity to humans. Representatives of *Clostridium*, *Phocaeicola*, *Cloacibacterium*, *Chrysobacterium*, *Brevundimonas*, *Sporacetigenium*, and *Sphingomonas* (Table 4) were found. Those species were identified mainly in samples from P4 in different conditions.

## 4. Discussion

The suitability of peats and sapropel from lakes for external usage in balneotherapy due to microbial contamination has been discussed for decades. One of the first studies performed in Austria concluded that peats have much better microbial quality results than those analysed from eutrophic lakes [38]. This was linked to the distance to the human settlements, which could impact microbial pollution that enters the studied environment. In 2006, the European Spas Association, during the General Assembly in São Pedro do Sul, adopted the Quality Criteria of the European Spas Association [39], highlighting that “the standards of hygiene governing the usage of a spa’s resources are based on legal and scientific principles”. The ISO 21426:2018 standard “Tourism and related services Medical spas” [18] specifies requirements for providing quality services at medical spas that use natural healing waters (except sea water) and other natural resources, such as peloids. For peloids, pathogenic microorganisms which are the cause of concern include *Pseudomonas aeruginosa*, *Staphylococcus aureus*, *Escherichia coli*, coliform bacteria, *Salmonella*, *Candida albicans*, *Aspergillus niger*. However, different EU countries have distinct regulations regarding indicator microorganisms in peloids and their contamination thresholds that are safe for external application (Table 1). In Lithuania, seven microbial indicators are used for peloid evaluation [17], considering only bacterial indicators, while ISO 21426:2018 indicate six bacterial and two fungal indicators. Using some of them as indicators for peloid quality assessment—mainly sulphite-reducing clostridia and colony-forming units—is questionable in Lithuania and other countries. 

All analysed ready-to-be-used peloid samples in our study had high quantities of sulphite-reducing clostridia, assessed using cultivation methods when there were supposed to be zero values. A similar situation was found in an earlier study from Lithuania [40]. The presence of sulphite-reducing clostridia in environmental samples, such as water or soil, is considered an indicator of faecal contamination of human or animal origin. Monitoring these bacteria is important for assessing the safety of water supplies and ensuring compliance with environmental regulations. However, considering peloid samples, more evidence shows that clostridia might be part of the environments from which peloids are sourced. Sulphite-reducing clostridia or *C. perfringens* assessment are not included in ISO 21426:2018 [18] for peloids, nor was it suggested by [37] or is used as an indicator under German regulations [6]. Historical analysis of lake sediment in Geneva revealed that the *Clostridium* proportionally increased in periods (1976–1987) when eutrophication intensified [41]. Still, the presence of this genus was also identified in the sediments layer from 1921. In thermal muds from Italy, anaerobic sulphite-reducing clostridia resulted in the most abundant group among assessed microbial parameters. Their growth was observed in 92.2% of samples, with CFU counts ranging from 3 to 2070 CFU/g [37]. In the thermal muds of France, *Clostridium* spp. was found in all the samples ranging from 10^1^ to 10^5^ CFU/g. Most of them belonged to *Clostridium bifermentans* and *C*. *sporogenes*, which are considered not to cause pathogenicity through the skin if it is not damaged [42]. Peloids are an anoxic matrix that might favour clostridia’s proliferation even though they are initially present in low quantities. Based on our long-read sequencing data, we found representatives of *Clostridiaceae* in the P3.I sample (20.3% of relative abundance). The tendency was that their relative abundance increased at 50 °C (44.2%) and also appeared in P4 at 40 °C and 50 °C when they were initially absent in this sample. ASV could be assigned to three species—*Clostridium tunisiense*, *C. bowmanni*, and *C. punence*. *C. tunisiense* was initially isolated from olive mill wastewater [43] and is known as a sulphite reducer. *Clostridium punense* was isolated from healthy human faeces [44] and is known for using sugar as a carbon source to generate acetic acid, butyric acid and hydrogen. Those species can grow at 43 °C with an optimum temperature of 37 °C [43,44].

Two samples (P3 and P4) reached the threshold of the number of colony-forming units set up in LT HN 126:2010 (<5.0 × 10^5^ CFU/g). In Germany, the thresholds are suggested to be <10^7^ CFU/g for mesophilic heterotrophic microorganisms determined at 20 °C and 36 °C [6]. Peat and lake sapropel contain a diverse and abundant microbial community that plays a crucial role in nutrient cycling, organic matter decomposition, and various other ecological processes. For example, depending on their condition in peatlands, the total viable bacteria ranged from 1.26 × 10^4^ CFU/g to 9.81 × 10^7^ CFU/g [45]. In the sapropel from five Latvian lakes, the number varied from 2.0 × 10^5^ to 2.3 × 10^7^ CFU/g [46]. Peloids are distinguished by their high microbiological activity, differentiating them from similar formations. Bacteria and fungi actively decompose organic residues, enriching therapeutic mud with humic and fulvic substances and gases. Continuous microbial activity stabilises volatile microcomponents like vitamins and enzymes in the mud. Thus, the total viable count serves as a general indicator parameter reflecting the overall microbial colonisation in the peloids, but it is hardly related to peloid contamination or specific pathogen species. 

Regarding peloid microbial diversity, previous research has focused on potentially pathogenic microorganisms [37,42], with some exceptions when the diversity of prokaryotes was analysed using both cultivation and molecular techniques. Using long-read metagenomic sequencing, we identified bacterial representatives from 23 different phyla and 149 taxa at the genus level. It is more than has been previously reported for similar environments by cultivation and in a range with the amount assessed with molecular methods. Twenty-six bacterial taxa were identified in therapeutical peat probes from Latvia using cultivation techniques [46]. In Ref. [19] at the family level, 40 taxons were identified in therapeutical peats and [47] in thermal waters and peloids at different stages of maturation, and up to 163 species were identified using short-read sequencing. Metagenomic sequencing and colony-forming unit, or most probable number counting, serve as valuable tools for assessing microorganisms, each presenting its own set of advantages and limitations. Metagenomic sequencing offers a comprehensive view of microbial communities, including both unculturable microorganisms (those that cannot be cultured using standard laboratory techniques) and viable but non-culturable (VBNC) microorganisms (those in a state that cannot be cultured). It allows species identification without the need for culturing and detecting novel or less-characterised microorganisms [48]. However, this method cannot differentiate between live and dead cells, limiting its ability to assess microbial viability. Additionally, it may face challenges in detecting low-abundance species, and the accuracy of results can be influenced by sequencing depth. Conversely, CFU counting provides a direct measure of viable microbial cells and allows for the isolation and study of culturable organisms, aiding in further characterisation. Nevertheless, it introduces bias as not all microorganisms can be cultured, leading to an underrepresentation of the true microbial diversity. This method exclusively measures viable cells, potentially underestimating microbial abundance if some cells are viable but non-culturable. Flow cytometry (FCM) or quantitative PCR (qPCR) can be employed to bridge this gap. qPCR can be used to assess VBNC bacteria by adding ethidium or propidium monoazide [49]. These compounds infiltrate damaged cell membranes, permeate deceased bacteria, and interact with the hydrocarbon segment of lifeless cell DNA, inducing structural alterations in the DNA. These modifications prevent the replication of dead cell DNA during the PCR reaction, allowing only the DNA from living cells to replicate. To address the above-discussed limitations, a comprehensive approach that combines different techniques—including sequencing for species identification, live/dead cell quantification using FCM or qPCR, and CFU enumeration, could provide a more precise and insightful evaluation of potentially pathogenic bacteria.

The microbiota in all four peloids was different. Proteobacteria dominated in the P1 and P2 peloid samples. In Ref. [47] identified the dominance of bacteria from the same phylum in peloids maturated using water from a thermal spring. From their identified taxa in our samples, representatives of *Rhizobiales* (P1.I, P1.40, P2.50) and *Burkholderiales* (all P1 and all P2) were also present, but they belonged to different genera.

The bacterial composition of the analysed peats (P3) sample was dominated by *Bacilli* class *Mesobacillus subterraneus* (BLAST KY202702, 99%), which was isolated initially from thermal springs, and *M. thioparans* (BLAST Nr_043762, 99.8%), initially isolated from continuous wastewater treatment culture system with a bacterial consortium [50]. *Haloimpatiens* of the *Clostridia* class of Firmicutes phylum were the next abundant. Generally, Firmicutes are widespread in environments mostly with diverse metabolic activity. However, that is an atypical community composition considering the source of peloid—peats. Using metagenomic techniques analysing therapeutic peats in Poland, the most predominant phyla were *Acidobacteriota* and *Proteobacteria*, which accounted for more than 75% of all samples and Firmicutes were not identified at all [17]. However, in culture-dependent microbiological methods in peat probes obtained in two Latvian balneotherapy spa sites [44], isolated species belonged to *Alpha*-, *Beta*-, and *Gammaproteobacteria*, *Actinobacteria*, *Clostridia*, *Bacilli* and *Flavobacteria*.

Bacteroidota was the most abundant in sample P4, with representatives of the *Rikenellacea* and *Flavobacteriaceae* families. The representative of *Rikenellaceae* had a similarity to uncultured bacteria isolated from paddy soil (BLAST AB700610.1), and the representative of *Flavobacteriaceae* had a similarity of 97.5% to uncultured bacteria isolated from wastewater treatment systems (BLAST LR637279). Even though the peloids were of sapropel origin, their microbial composition from P1 and P2 might differ due to premixing with mineral water before use. We identified *Polaromonas*, *Rhodoferax*, *Curvibacter* and *Caulobacter* genera, which were previously defined as consisting of a significant part of mineral water microbiota [51,52]. Only in this sample did we identify *Thiobacillus* (*Thiobacillus thioparus* and *T. thiophilus*), also found by [53] in thermal water and mud of an Italian spa. *Thiobacillus* there comprehended sulphur-oxidising bacteria and were highly represented in the thermal water but reduced during mud maturation. In P4, we also found *Sulfuricurvum kujiense*, sulphur-oxidising bacteria. *Poloromonas*, *T. thioparus* and *Sulfuricurvum* constituted a significant part of healing mud in Croatia [54], contributing to the sulphur cycle by oxidising reduced-sulphur compounds. *Polaromonas* is known for detoxifying the environment by removing heavy metals [11], and their presence might improve the medicinal properties of mud.

However, we also found taxa related to faecal or sewage environments in this sample. Using cultivation, we found *E*. *coli*, and using sequencing, we identified *Clostridium punance* and *Cloacibacterium normanensee*.

Considering previously identified microorganisms from therapeutic muds and thermal mineral waters with potential activity on skin microbiome [8], we could locate only *Cyanobacteria* in low relative abundances in sapropel peloids (P1 and P2). However, some bacteria found in our samples are known to play a crucial role in the properties associated with peloid formation. This includes their involvement in processes such as the creation of humic substances or participation in the sulphur cycle.

The large variety of bacteria (in the class *Alphaproteobacteria* and *Betaproteobacteria*, *Actinobacteria*, *Firmicutes*, or *Bacteroidetes*), depending on the source, can degrade humic substances, which are important and valuable components of peloids. Certain genera found in our samples are considered humic substance degraders [55,56,57], and their presence might indicate the significant presence of humic substances. Indeed, in our samples we found *Sphingobium* (P4.50), *Acidovorax* (P2.1, P2.40), *Chrysobacterium* (P4.I, P4.40, P4.50), and *Clostridium* (P3.I, P4.40, P4.50). *Bacillus*, which recently was suggested to be split into six new genera [58], also degrades humic substances; *Mesobacillus* and *Peribacillus* in high abundances were identified in samples P3.I and P3.50. In freshwater wetlands, especially peatlands, where organic macromolecules accumulate due to acidic and anoxic conditions, most dissolved organic matter is represented by redox-active humic substances, including humic acids that are highly reactive towards Fe. Adding peat-derived humic acid extract supported *Sideroxydans lithotrophicus* proliferation [59]; we identified this species in P4.I, P4.40, and P4.50 of sapropel origin.

Our tested temperature conditions, 40 °C and 50 °C, impacted the shift in the abundance of specific microorganisms or relative abundance within the microbial community of peloids. As we see from our data, the different temperatures had different effects on cultivable microorganisms. In sample P2, the coliform amount stayed the same despite different temperature conditions, while in sample P4—it decreased at 50 °C. A very different situation was observed with *E*. *coli* when the samples were heated; in P2, it decreased, and in P4, it increased, especially at a temperature of 40 °C. This indicates that different microorganisms or species might be affected differently by different temperature conditions, and preheating peloid samples in the temperature range from 50 to 55 °C might not be enough to prevent unwanted microorganisms. A previous study showed that using the Colilert-18, up to 45 species of coliform bacteria belonging to 20 genera could be identified [60], which could behave very differently concerning temperature conditions. [37] in his study found that depending on mud type, *E. coli* was found at 39.6 °C and absent at 46 °C, while total coliforms were present in the range of 39.6–47.2 °C, while absent in the range of 48.1–58.3 °C. They suggested that the sterilisation step with thermal water ≥ 60 °C should be compulsory just before treatment to ensure mud hygienic quality. However, more studies using different peloid types and temperature regimes should be performed to understand the topic better. Decimal reduction doses for microorganisms in peat samples and radiation sterilisation doses of peat for the gamma and electron beam radiation were determined by [46]. The highest radiation resistance was observed for *Bacillus mycoides.* However, heat sterilisation carried out in a steam autoclave at 121 °C for 15 min proved to preserve the peat sterols better than radiation sterilisation, which may lead to different therapeutic effects in their application. Despite our metagenomic sequencing efforts, we did not detect the presence of *Enterobacterales*, the order to which coliform bacteria, including *Escherichia coli*, belong. Although we identified these bacteria through cultivation, they seem to constitute only a minor fraction of the community, which was not amplified during the sequencing. This phenomenon was also found during other investigations on coliform presence in the environment [60].

Considering the metagenomic data analysis, the temperature has affected the composition shift and the increased abundance of certain species. The sample P1.40 had the highest microbial diversity, with representatives of the *Bacillaceae*, *Rikenellaceae*, *Clostridiaceae*, *Desulfocapsaceae*, *Gallionellaceae*, *Woykebacteria*, *Beijerinckiaceae*, *Nitrospiraceae*, *Beggiatoaceae*, and *Methylophilaceae* comprising more than the 50% of the observed taxa, but leaving space for other families not belonging to the dominant classes, such as representatives of the families *Gemmatimonadaceae*, *Phycisphaeraceae*, or *Xanthobacteraceae*. Chloroflexi found in none of the initial samples after preheating was found in samples P1 and P2 at both temperature regimes. In thermal muds examined by [61], Chloroflexi revealed an increase in relative abundance at higher temperatures (>45.7 °C) too. Some Chloroflexi species are anaerobic phototrophs, meaning they can perform photosynthesis without oxygen. This adaptation is often found in environments with elevated temperatures, such as hot springs or thermal vents. Another example is a *Methylocytis*, which was not determined at initial conditions in samples P1 and P2 but was found under increased temperature conditions at 40 °C and 50 °C. Reference [62] reported that due to rising temperatures, there could be significant increases in the relative abundances of methanotrophs such as *Methylocystis*. Elevated temperatures can promote biochemical processes in peloids, in this case, methane production, and methanotrophs may respond by showing heightened enzymatic activity and improved metabolic efficiency. This enhanced activity enables them to utilise methane as a substrate more effectively. Furthermore, increased temperatures can impact the competitive interactions among various microbial species. Another species, *Mesobacillus*, was also found in high abundance (sample P3, 41.8% of relative abundance). In [63] study, these bacteria were cultured at 55 °C and 60 °C. Different bacterial taxa’s varying thermal preferences and tolerances might influence the changes in peloid bacterial populations under specific temperatures. Some bacteria are more adaptable and thrive in a broader temperature range, allowing them to flourish as temperatures change. On the other hand, specific bacterial populations may remain unchanged because they are better suited to the existing temperature conditions and are less affected by fluctuations. Factors such as metabolic activity, enzyme function, and overall physiological responses to temperature can vary among bacterial taxa, contributing to the observed changes or stability in populations under specific temperature conditions.

## 5. Conclusions

This pilot study on microbial diversity and dynamics of peloids (sapropel and peats) used in spa procedures in Lithuania revealed different microbial communities. Under tested peloid heating temperatures of 40 °C and 50 °C, the bacterial community shifted in peloids of sapropel origin, and, at higher temperatures, the relative abundance of specific taxa increased. The higher temperature might affect the communities participating in the sulphur cycle and humic substance degradation, the peloid properties that might be important for human use. However, how it changes still needs to be revealed. Using cultivation and long-read sequencing data in some samples, we identified bacteria that might be potential pathogens or linked with faecal sources. Additional research is necessary to reveal the stages at which they might enter the system, particularly when multiple steps in the preparation of peloids are involved. Moreover, the temperature regime suggested for safety assurance before peloid use in the range from 50 to 55 °C is too low to prevent the potentially pathogenic bacteria if they are present in peloids. Further detailed analysis is necessary to comprehend the various stages of peloid preparation before their utilisation in spa procedures about microbiota. This is essential for balancing beneficial and non-beneficial microbiota and their related properties.

## Figures and Tables

**Table 1:** Peloid properties.

Sample	Sample Taken	Sample Type	Organic Matter %	Decomposition Rate %
P1.I	From 5-litre plastic containers	Sapropel (brown)	81.9	75.9
P2.I	From 10-litre plastic containers	Sapropel (brown)	91.7	79.4
P3.I	From container	Peat	70.7	81.4
P4.I	Premixed with mineral water, kept in a plastic container	Sapropel (white)	14.3	100

**Table 2:** The microbiological parameter values of peloids used in this study and the thresholds of parameters indicated in different literature sources. Microbial parameters were assessed by the National Public Health Surveillance Laboratory. Additional microbiological parameters that are supposed to be estimated in peloids: according to ISO 21426:2018 [18]—coliform bacteria, fungi (*Candida albicans*, *Aspergillus niger*); according to [37]—dermatophytes; for Germany [6]—yeasts and fungi, <10^4^ CFU/g; coliforms, <10^4^ CFU/g.

		Worm Eggs, Units/kg	*E*. *coli* Number, CFU/g	Sulphite-Reducing Clostridia, 1 g	*Salmonella*, 25 g	*Staphyloccocus aureus*, g	*Pseudomonas aeruginosa*, g	Colony Forming Units, g
**Sample ID**	P1	0	<10	**3.3 × 10^3^**	0	0	-	5.6 × 10^4^
	P2	0	<10	**2.2 × 10^2^**	0	0	-	7.5 × 10^4^
	P3	0	<10	**2.1 × 10^2^**	0	0	-	**9.2 × 10^7^**
	P4	0	<10	**4.1 × 10^1^**	0	0	-	**8.4 × 10^6^**
**Microbial parameters and thresholds in different literature sources**	LT Hygiene norm 126:2010	0	<100	0	0	0	0	<5.0 × 10^5^
	ISO 21426:2018	*	100/100 ml	*	0	0	0	at 22 °C, no threshold provided
	[35]	*	0	*	*	0	0	*
	[6] (Germany regulation)	*	<10^2^ CFU/g	*	*	0	0	at 20 °C, <10^7^ CFU/g;
								at 36 °C <10^7^ CFU/g

- not evaluated. * not included as an indicator. he ones reaching the thresholds defined by LT Hygiene Norm 126:2010 [17] are noted in bold.

**Table 3:** The microbiological parameter values of peloids in the experiment with different temperature preheating.

	Total Coliform, Average MPN/g			*E*. *coli* Number, Average MPN/g			*Candida albicans*, Average CFU/g			*Aspergillus niger*, Average CFU/g		
Sample ID	Initial	40 °C	50 °C	Initial	40 °C	50 °C	Initial	40 °C	50 °C	Initial	40 °C	50 °C
P1	0	0	0	0	0	0	0	0	0	0	0	0
P2	4.2	9.3	3.6	0.3	0	0.1	0	0	0	0	0	0
P3	0	0	0	0	0	0	0	0	0	0	0	0
P4	101.1	85.1	27.1	0.2	2.2	1	0	0	0	0	0	0

**Table 4:** The presence of bacterial taxa that could potentially be pathogenic or are linked to faecal contamination in peloid samples.

Genus	Species	BLAST No	Similarity	BLAST Source	Additional Information	Sample	Relative Abundance%
*Clostridium*	*tunisiense*	NR_115161	98.54%	from an olive mill wastewater		P3.I	1.98
						P4.40	1.08
						P4.50	0.40
	*bowmanii*	NR_114765	98.08%	mat sample from the lake		P4.40	0.48
						P4.50	0.89
	*punense*	NR_145903	99.09%	healthy human faeces		P4.40	0.36
						P4.50	0.20
*Phocaeicola*	*dorei*	CP046176	99.93%	from human		P3.I	0.27
*Cloacibacterium*	*normanense*	CP034157	99.93%	untreated municipal wastewater		P4.I	6.08
						P4.40	2.63
						P4.50	2.97
*Chryseobacterium*	alike *faecale*	CP087583	96.25%	from faeces		P4.I	1.6
						P4.40	0.23
						P4.50	0.6
*Brevundimonas*	*bullata*	NR_113611	99.21%	from soil	Some *Brevundimonas* species are considered potential human pathogens	P4.I	0.25
*Sporacetigenium*	*mesophilum*	NR_043101	99.37%	from an anaerobic digester treating municipal solid waste and sewage		P4.40	1.31
						P4.50	0.20
*Sphingomonas*		NR_148321	96.60%	from activated sludge	Some species are known as human pathogens	P4.50	0.20
*Listeria*	*monocytogenes*	LT906436	100%	isolated from rabbit	Pathogenic bacteria that causes the infection listeriosis	P2.I P3.50	2.1 0.5

## Data Availability

The data presented in this study are available upon request from the corresponding author.

## Supplementary Materials

The following supporting information can be downloaded at: https://www.mdpi.com/article/10.3390/ijerph21030335/s1, Figure S1: Rarefaction curves of the bacterial 16S rRNA gene sequences from the 11 based on ASV calculated at 99% identity. The total number of sequences analysed is plotted against the number of ASVs observed in the same community; Table S1: Taxonomy table.
