# Supplementary figures and images for: Microbial Composition Dynamics in Peloids Used for Spa Procedures in Lithuania: Pilot Study
Source: Int J Environ Res Public Health. 2024 Mar 12;21(3):335. doi: 10.3390/ijerph21030335 (PMC10970318; doi:10.3390/ijerph21030335)

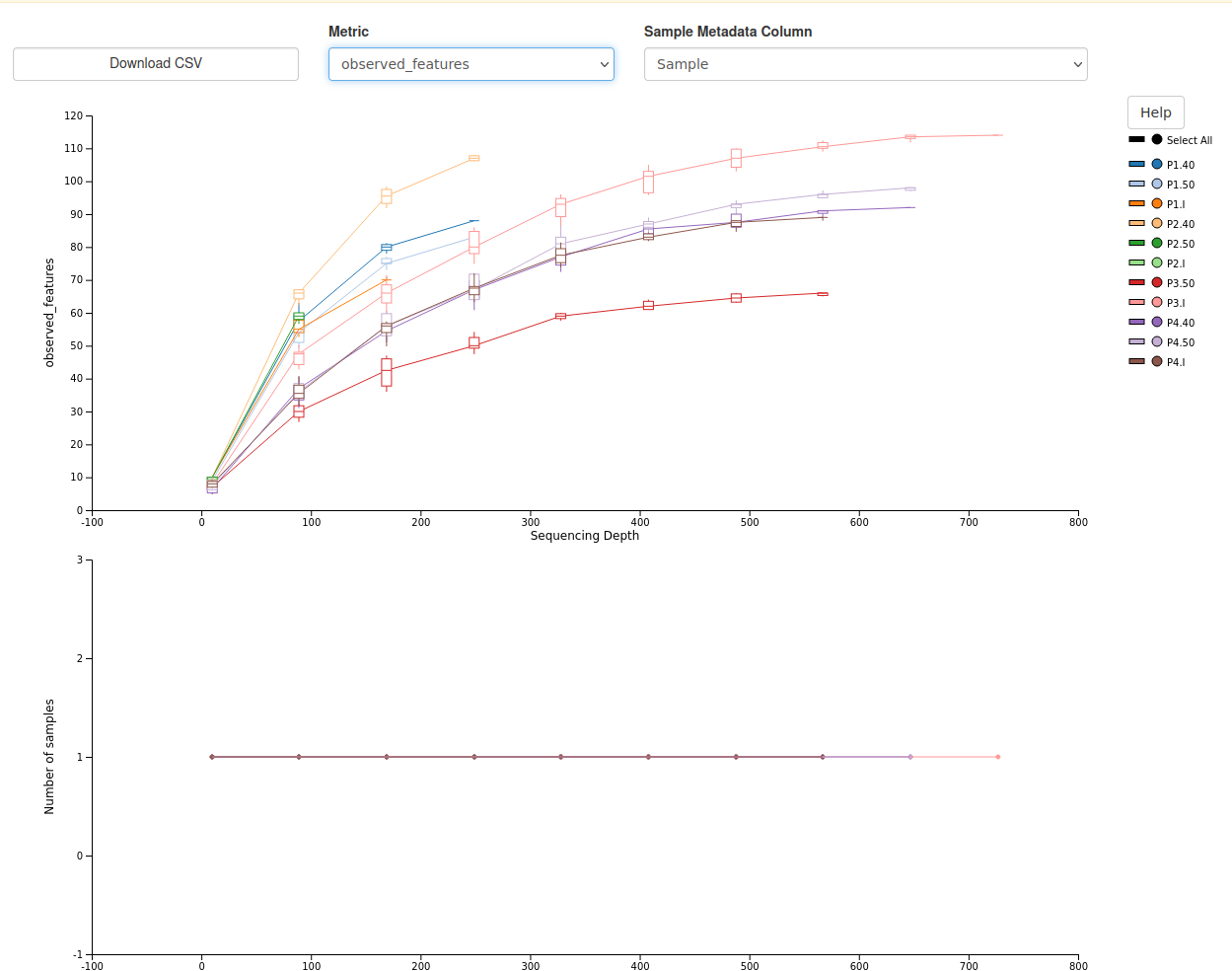

Supplement: Supplementary file 1 [file ijerph-21-00335-s001.zip › Fig. S1.jpg]
